# Isolated diaphragmatic metastasis from periosteal osteosarcoma of the humerus: a case report

**DOI:** 10.1186/s13019-020-01103-4

**Published:** 2020-04-16

**Authors:** Luo Zhao, Yingzhi Qin, Dongjie Ma, Wenze Wang, Shanqing Li, Hongsheng Liu

**Affiliations:** 1 Department of Thoracic Surgery, Peking Union Medical College Hospital, CAMS & PUMC, No 1 Shuaifuyuan, Beijing, 100730 China; 2 Department of Pathology, Peking Union Medical College Hospital, CAMS & PUMC, Beijing, 100730 China

**Keywords:** Periosteal osteosarcoma, Diaphragm metastasis, Gene mutation, Case report

## Abstract

**Background:**

Isolated diaphragmatic metastasis is rarely associated with periosteal osteosarcoma.

**Case presentation:**

A 24-year-old female patient was found to have periosteal osteosarcoma of the right humerus 11 years ago. Computed tomography showed a mass in her left chest in 2018, and thoracotomy was performed to remove the tumour. The tumour showed the same characteristics as the original periosteal osteosarcoma. Genetic analysis of the tumour sample showed a TP53 point mutation and CCNE1 gene copy number variants.

**Conclusions:**

We believe this is the first case report of haematogenous metastasis to the diaphragm of periosteal osteosarcoma, and an investigation of the genetic factors may help to unravel the underlying cause of periosteal osteosarcoma.

## Background

Periosteal osteosarcoma (PO) is an uncommon primary malignant bone tumour that represents less than 2% of all osteosarcomas. Compared with conventional osteosarcoma, periosteal osteosarcoma presents less aggressive biological behaviour and a lower tendency to metastasize. We herein report an unusual case of isolated diaphragmatic metastasis that originated from periosteal osteosarcoma 11 years later.

## Case presentation

In 2008, a 24-year-old non-smoking female was admitted to the hospital for right upper limb pain. On examination, she appeared to have a tender fusiform bony-hard swelling of the right upper arm. An X-ray scan showed an extraosseous tumour on the periosteal surface mainly in the mid humerus. A biopsy of the lesion was performed, with the histological result confirming periosteal osteosarcoma diagnosis. Computed tomography (CT) of her thorax and emission computed tomography (ECT) scan demonstrated no metastases. Segmental resection of the proximal periosteal osteosarcoma with internal fixation grafting was performed. The histology report demonstrated the presence of periosteal osteosarcoma. The report indicated that the local excision was complete with wide excision margins. She received adjuvant therapy postoperatively with 4 cycles of a cisplatin and doxorubicin regimen.

**Fig. 1:**
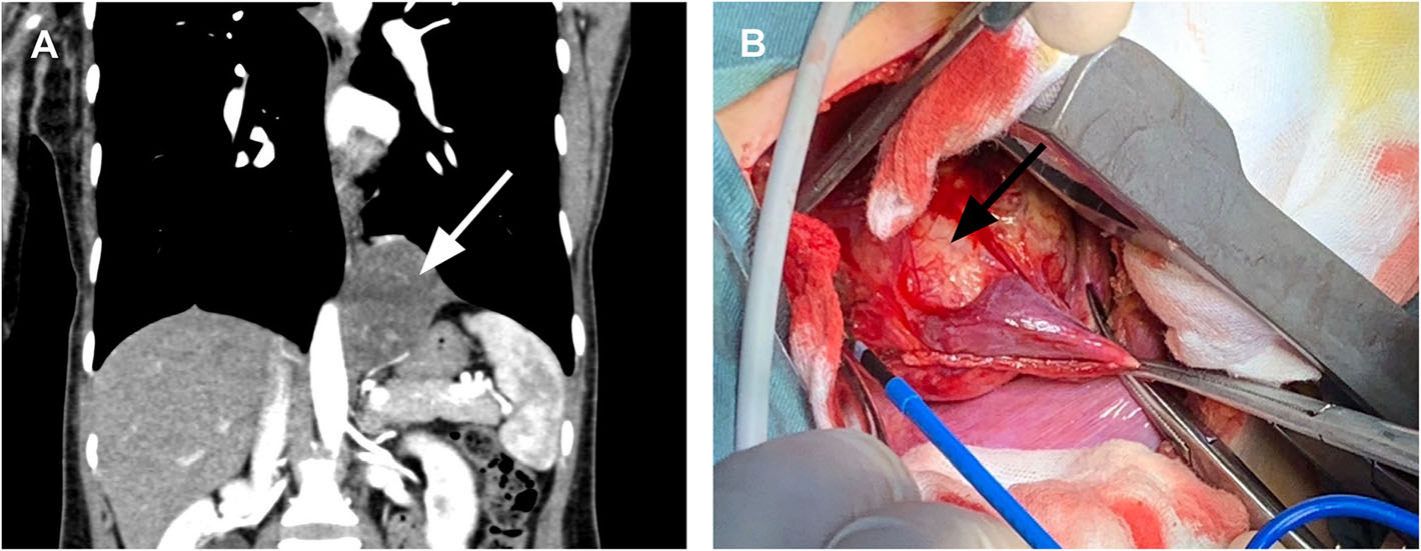
**a** CT images showing the tumour (white arrow) was enhanced and was found to be closely related to the oesophagus and diaphragm. **b** Intraoperative photographs showing the tumour (black arrow) originating from the diaphragm and adhering to the left lower lung

**Fig. 2:**
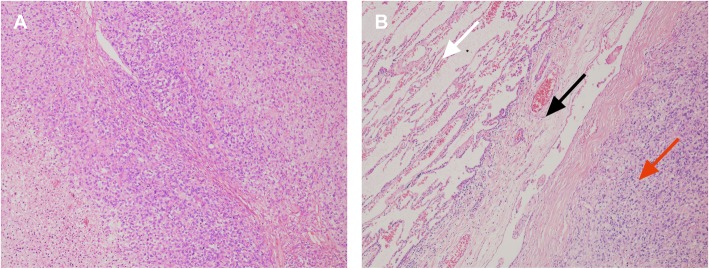
Pathological findings. **a** Periosteal osteosarcoma cells (haematoxylin-eosin staining, 150×). **b** The tumour did not invade the lung tissue (haematoxylin-eosin staining, 60×; white arrow, pulmonary tissue; black arrow, pleura; red arrow, periosteal osteosarcoma)

We followed the patient every 6 months with routine complete blood counts and liver function, serum alkaline phosphatase, and lactate dehydrogenase level tests, along with X-rays of the right humerus. ECT and CT scans of the chest were performed annually. No signs of recurrence were found through these examinations. However, chest CT found a mass in her left chest for the first time in December 2018. The mass was 5.5 cm, and the mass increased to 6.1 cm half a year later (Fig. 1a). The patient underwent thoracotomy in June 2019. We found a solitary mass on her diaphragm during the operation. The mass partially involved the left lower lung without involving any other organs, including the oesophagus or chest wall (Fig. 1b). We removed the tumour that was partially on the left lower lung with sufficient tumour resection margins and repaired the diaphragm defect. The patient was discharged 4 days after surgery. Postoperative histopathology showed a tumour measuring 6 cm. Postoperative pathology revealed that the tumour was mainly distributed in the diaphragm muscle tissue, and the pulmonary membrane of the left lower lung was not invaded (Fig. 2). The tumour showed an intermediate grade periosteal osteosarcoma, the same characteristic as the original tumour in the right humerus. Genetic analysis of the tumour sample showed a tumor protein 53 (TP53) (exon 8) p.R280K point mutation and Cyclin E1 (CCNE1) gene copy number variants (CNVs). The patient received chemotherapy after surgery. No severe adverse effects appeared, and no signs of recurrence were found at the 6-month follow-up visit.

## Discussion and conclusions

Osteosarcoma is a primary, high-grade malignant tumour. The periosteal osteosarcoma subtype of osteosarcoma originates from the periosteum without evidence of medullary involvement. A 10-year overall survival rate of 84% and a 10-year event-free survival rate of 52% have been reported [1]. According to the current literature, the local recurrence rate is 5.6–40%, and the metastatic rate is 11.1–22.2%, of which lung metastasis and pleural metastasis are the most common [2]. Isolated diaphragmatic metastasis of PO has not been reported in the English literature. Most patients had distant recurrences, which is different from classic osteosarcoma. Genuine haematogenous metastasis to the diaphragm is extremely rare. Complete tumour resection is the main treatment for periosteal osteosarcoma, but the effect of chemotherapy is still controversial. Due to the rarity of periosteal osteosarcoma, there is no evidence-based study for this treatment. According to the current literature, periosteal osteosarcoma is less aggressive, and the main treatment is complete resection of the original tumour. For metastatic lesions, such as lung metastasis of colon cancer, palliative and complete resection of the lesions is usually the recommended treatment. In our opinion, a negative surgical margin is enough for these patients. During the operation, we found the tumour capsule intact and clear, so we removed the diaphragm 1 cm from the tumour and partial left lower lung 3 cm from the tumour. Postoperative pathology also confirmed that the margin was clean. Certainly, we also recommend intraoperative frozen pathology to ensure a negative surgical margin, but this may be difficult in practice. If a positive surgical margin was found after surgery, palliative radiotherapy should be considered. Unfortunately, the rarity of this disease does not allow such a study to be conducted. Only randomized studies can answer the question of whether chemotherapy is beneficial for these patients.

Genetic factors may be a novel approach for further research in periosteal osteosarcoma. Maheshwari et al. found the TP53 gene (exon 8) mutation in two PO patients [3]. In our patient, genetic analysis of the tumour showed a TP53 (exon 8) p.R280K point mutation, and CCNE1 gene copy number variants (CNVs). TP53, a tumour suppressor in the DNA damage pathway, is the most common mutated gene in oncogenesis [4]. TP53 is regulated by MDM2, which in turn mediates Wnt signalling, which itself plays an important role in the aggravation of osteosarcoma tumorigenesis. The TP53 (p.R280K) mutation has been recorded in the COSMIC database, and many are found in the urinary tract, in addition to breast, skin, lung and thyroid cancers. Most of the mutations (70–80%) are missense point mutations, most of which affect the DNA binding domain (exons 5–8). Clinical studies have confirmed that 95.1% of TP53 mutations in tumours mainly occur at the highly conserved sites 175, 245, 248, 249, 273, and 282. Researchers have confirmed that mutations in this gene can lead to more aggressive tumours and poor prognosis. However, at present, the Food and Drug Administration has not approved any targeted drugs for TP53. Gene therapy, targeted cancer vaccines, and anticancer drugs for the TP53 mutation are still undergoing clinical trials. The CCNE1 gene encodes cyclin E1, and its copy number variation may lead to the overexpression of cyclin E1, promote cell cycle progression, induce many cancers, and indicate poor prognosis [5]. Thus, these two possible genetic factors may affect the oncogenesis of periosteal osteosarcoma.

There has been very little research or case reports describing the details of recurrence. We believe this is the first case report of haematogenous metastasis to the diaphragm of periosteal osteosarcoma. Wide surgical removal of the tumour is the best treatment to cure the disease. Chemotherapy treatment plays a controversial role in improving the survival rate for these patients. Genetic factors must be examined to unravel the underlying cause of PO during initial phases of diagnosis and care.

## Data Availability

The datasets supporting the conclusions of this article are included within the article and its additional files.

## Abbreviations

PO: periosteal osteosarcoma
CT: Computed tomography
ECT: Emission Computed Tomography
TP53: Tumor Protein 53
CCNE1: Cyclin E1
CNV: Copy number variants
DNA: Deoxyribonucleic acid
